# Role of the subthalamic nucleus in perceiving and estimating the passage of time

**DOI:** 10.3389/fnagi.2023.1090052

**Published:** 2023-03-02

**Authors:** Motoyasu Honma, Fuyuko Sasaki, Hikaru Kamo, Maierdanjiang Nuermaimaiti, Hitoshi Kujirai, Takeshi Atsumi, Atsushi Umemura, Hirokazu Iwamuro, Yasushi Shimo, Genko Oyama, Nobutaka Hattori, Yasuo Terao

**Affiliations:** ^1^Department of Medical Physiology, Kyorin University of School of Medicine, Tokyo, Japan; ^2^Department of Neurology, Juntendo University School of Medicine, Tokyo, Japan; ^3^Department of Neurosurgery, Juntendo University School of Medicine, Tokyo, Japan; ^4^Department of Neurology, Juntendo University Nerima Hospital, Tokyo, Japan

**Keywords:** Parkinson’s disease, subthalamic nucleus, deep brain stimulation, temporal sense, representation

## Abstract

Sense of time (temporal sense) is believed to be processed by various brain regions in a complex manner, among which the basal ganglia, including the striatum and subthalamic nucleus (STN), play central roles. However, the precise mechanism for processing sense of time has not been clarified. To examine the role of the STN in temporal processing of the sense of time by directly manipulating STN function by switching a deep brain stimulation (DBS) device On/Off in 28 patients with Parkinson’s disease undergoing STN-DBS therapy. The test session was performed approximately 20 min after switching the DBS device from On to Off or from Off to On. Temporal sense processing was assessed in three different tasks (time reproduction, time production, and bisection). In the three temporal cognitive tasks, switching STN-DBS to Off caused shorter durations to be produced compared with the switching to the On condition in the time production task. In contrast, no effect of STN-DBS was observed in the time bisection or time reproduction tasks. These findings suggest that the STN is involved in the representation process of time duration and that the role of the STN in the sense of time may be limited to the exteriorization of memories formed by experience.

**Figure:**
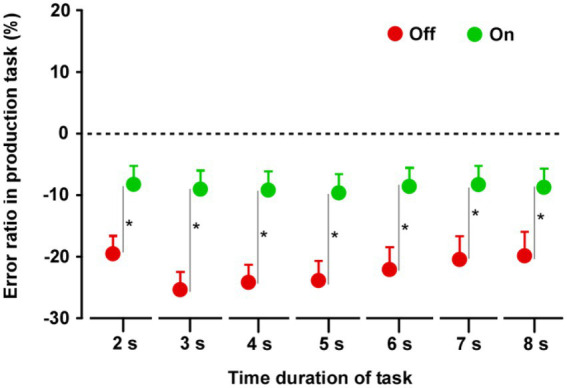
GRAPHICAL ABSTRACT

## Introduction

Subjective sense of time (temporal sense) is essential for perceiving and estimating the passage of time in daily life. Although the temporal sense is influenced by many factors, including circadian rhythms, emotion, and disease (Hancock et al., 1992; Honma et al., 2016; Mella et al., 2019), we previously showed that temporal sense is robust in each individual and consolidated at a stable value under certain conditions (Honma et al., 2021). This also holds for patients with Parkinson’s disease (PD) in whom this is also robust and resistant to change; in the presence of dopamine deficiency, accurate time duration learned by feedback training quickly returns to inherent underestimated levels, and even after applying repetitive transcranial magnetic stimulation over the prefrontal cortex for inducing cortical plasticity and manipulating/consolidating time sense (Honma et al., 2022). This shortened temporal sense or memory representation may be likened to the shuffling gait in PD patients, with steps gradually asymptoting to a smaller level in the absence of an external cue.

Details of temporal processing for the sense of time or its neural correlate, however, have not been clarified. Unlike light and sound, time does not have a dedicated sensory organ. Temporal processing may be mediated not by a single or a few brain areas but by a complex network involving multiple brain regions (Buhusi and Meck, 2005; Shi et al., 2013), including the prefrontal cortex, striatum, and subthalamic nucleus (STN) (Nani et al., 2019), but the precise role of individual brain regions remains unresolved. While the brain regions involved in temporal processing largely overlap with those for motor control and sensory perceptual processing, regions serving memory, in this case, the temporal representation of time, may be also involved, consistent with the view that time and memory are closely interlinked (Teki et al., 2017).

Psychologically, the scalar expectancy theory (SET) postulates that temporal sensory processing comprises different processes, including clock (pacemaker, switch, and accumulator), memory (short-term and reference memory), and decision stages (Gibbon, 1977; Gibbon et al., 1984). It also assumes that different mechanisms may serve different temporal processes, e.g., mechanisms of representation and perception of time. The time production task reflects a function to read out time duration in reference to the representation of time duration (reference memory); the representation of time refers to the sense of time or a kind of “time scale” acquired through what one has experienced and stored in long-term memory as a scale for reference (for example, the time scale for subjective 10-s duration is established by repeated experience of the physical 10-s duration) (Baudouin et al., 2006; Jozefowiez and Machado, 2013). In contrast, the time bisection task reflects perceptual function, a process of perceiving and recognizing the current time with respect to the subjective sense of time learned shortly in advance (Atakan et al., 2012; Ogden et al., 2018). Meanwhile, time perception involves inputting physical time duration into the short-term memory to recognize its duration. In SET, temporal information processing is considered a cognitive process coordinating time perception and memory across a wide range of memory processes both utilizing the internal clock, and the abnormality in temporal production and perception has been explained by the abnormal pace of the internal clock. This may be addressed by evaluating time processing and perception functions at the same time, but few studies have studied both simultaneously in the same study.

PD patients, in whom basal ganglia dysfunction with dopamine deficiency likely causes bradykinesia (slowness of movements), have pronounced deficits in temporal processing compared with normal participants (Smith et al., 2007; Honma et al., 2017, 2018). Slowness may also involve the mind’s temporal processing. The basal ganglia have been postulated to set the pace of the “internal clock.” If we postulate that the mind uses an internal clock ticking at a regular rate to perceive the passage of time, it would tick more slowly in dopamine deficiency. Earlier studies have provided evidence consistent with the slowed clock hypothesis, which states that dopamine deficiency slows down the pace of the internal clock, which is corrected by dopaminergic medication improving the estimation of duration in the time production task in PD patients (Pastor et al., 1992; Lange et al., 1995; Smith et al., 2007; Koch et al., 2008; Wild-Wall et al., 2008). However, later studies have not necessarily supported this view. For example, in the time production task, PD patients evaluate (produce a specified time duration) the subjective time duration as shorter than normal participants (Honma et al., 2016). When PD patients estimate the duration of the period that a figure is visible on a screen as shorter or longer relative to two standard durations, PD patients are more likely to judge the duration as longer compared with healthy participants (Zhang et al., 2016). The pace of the internal clock can also be studied by the synchronized tapping task, which requires participants to press a button or tap a keyboard in synchrony with repetitive tones presented at fixed intervals (synchronization task, S) and to continue tapping at the same pace after the tones have been removed (continuation task, C), have found inconsistent results, reporting the pace of the internal clock to be faster (Ivry and Keele, 1989; O'Boyle et al., 1996; Harrington et al., 1998; Jones et al., 2011), slower (Pastor et al., 1992), or unchanged (Duchek et al., 1994; Spencer and Ivry, 2005; Wojtecki et al., 2011; Joundi et al., 2012) relative to normal participants. While temporal processing deficits in parkinsonism remain to be characterized, dopamine deficiency may not be the sole mechanism leading to the various temporal processing deficits in PD patients. Finally, in terms of the sense of time, some PD patients exhibit short production of duration compared to actual time, indicating a “faster” flow of time (Honma et al., 2018). Many findings are thus difficult to explain simply by the slowed clock hypothesis (Terao et al., 2021), and revision of the SET view should be considered. Additionally, because dopaminergic medication operates on various brain regions, it is difficult to verify the role of each region(s) alone play a critical role in temporal sensory processing and how (the cause-and-effect relationship) (Pastor et al., 1992; Nani et al., 2019).

Recently, deep brain stimulation (DBS) of the subthalamic nucleus (STN), playing a physiologically pivotal role in the pathomechanism of PD (Nambu, 2004; Wichmann and Soares, 2006), has come to be used widely for reducing PD patients’ motor symptoms (Sasaki et al., 2021; Tai, 2022). The inconsistent findings regarding dopamine deficiency and the pace of the internal clock can be addressed by manipulating the function of the STN, providing novel insights into temporal processing in terms of the internal clock and temporal sensory processing (temporal sense). STN-DBS also affects cognitive functions (Oyama et al., 2011; Tokushige et al., 2018) by altering the function of the basal ganglia-thalamo-cortical loop (Santaniello et al., 2012). A study of the effect of STN-DBS on temporal sense in PD showed that STN-DBS had no significant effect on perceptual timing in the hundreds of milliseconds range, unlike its effect on motor symptoms (Cope et al., 2014). STN-DBS also has a significant effect on the time reproduction task to measure the ability of short-term memory unrelated to internal clock (Koch et al., 2004). However, it is unclear whether STN-DBS affects temporal sense in the few seconds range, in which memory and other factors are likely to interact.

Animals and humans can process different ranges of timescales, ranging from microseconds, milliseconds, seconds to minutes, and a day (circadian rhythms), and it has been suggested that the neural structures responsible for temporal processing differs for these different time rages (Merchant and de Lafuente, 2014). Although these systems for different timescales may all contribute to the formation of the sense of time, in this study, we focused on the time scale of seconds to minutes range, which is considered to be closely associated with and processed within the motor system such as the basal ganglia and the cerebellum. We investigated the role of the STN in temporal sense processing by looking at what happens when the DBS device is switched on/off in PD patients receiving STN-DBS.

Three temporal processing tasks have been widely used to address distinct aspects of time perception. In the production task, subjects produce the duration of time instructed verbally, according to time scale formed by experienced and stored in memory, but does not require the ability to discriminate different time durations; in the reproduction task subjects are asked to reproduce the presented duration, for which it is neither required that the time scale stored in reference memory or that the ability to discriminate different duration is normal. In the bisection task to ask subjects whether the immediate duration of time presented is longer/shorter compared to the immediately preceding one (discrimination between different durations), whereas it does not depend on whether or not the reference duration formed by experience and stored in memory is normal.

By comparing performance of temporal cognitive tasks, we investigated whether STN DBS affects the ability referring to time duration formed by experience and stored in memory, the ability to discriminate different durations, of the ability to reproduce different durations, or any combination thereof. We predicted that performance is improved in time production task if DBS-STN affects the ability of reference duration formed by experience. Alternatively, if DBS-STN affects the ability to discriminate differences of duration, performance should be improved in the time bisection task. Finally, if DBS-STN affects the ability to reproduce duration, performance is improved in time reproduction task.

## Materials and methods

### Participants

This study was approved by the ethics committee of Juntendo University School of Medicine and conducted according to the principles of the Declaration of Helsinki (identifier: 18-215). This study was registered in the University hospital Medical Information Network (UMIN)-CTR (ID: UMIN000033776, 20/08/2018). All patients provided written informed consent before the experiments. G*Power (Version 3.1.9) specified that a sample size of 27 would be needed to obtain 70% power to detect a medium effect with an alpha of 0.05. Effect size (0.50) was determined by previous researches using temporal task (Honma et al., 2016, 2017, 2018, 2021, 2022; Terao et al., 2021).

There were 28 PD patients with an implanted DBS device (4 women and 24 men; mean age: 62.7 years, range: 51–74 years). The average duration of illness was 14.5 ± 3.7 years. All patients were right-hand dominant. PD severity was measured using the Unified Parkinson’s Disease Rating Scale-part III (Martinez-Martin et al., 1994) (average: 19.1 ± 7.8). We also examined general cognitive functions using the Mini-Mental Status Examination (Folstein et al., 1975) (28.6 ± 1.3) and Montreal Cognitive Assessment (Nasreddine et al., 2005) (26.8 ± 2.7). The neurologist diagnosed that none of the participants had dementia. All patients were tested for dopamine transporter (DaT) activity using DaT imaging (Kagi et al., 2010). The radioactive agent bound to DaT was expressed using a specific binding ratio, which is the ratio of the radiation in the striatum to those in the whole brain, calculated by the Bolt method (Tossici-Bolt et al., 2006). The average value of DaT was 1.78 in total (range: 0.11–4.64), and 1.83 in the right (0–4.83, SD = 1.8), and 1.72 in the left (0.22–4.44, SD = 1.7). Parkinson’s disease-related medications were discontinued at least 12 h before the tests were performed. Some subjects also had comorbid symptoms or diseases other than PD (Supplementary Figures).

All patients underwent surgery for bilateral implantation of stimulation electrodes (Model 3,389, Medtronic, Minneapolis, MN, United States) in the STN (13 patients, Vercise Gevia, Boston Scientific, Boston, MA, United States; 3 patients, Vercise PC, Boston Scientific, Boston, MA, United States; 7 patients, Vercise Genus RC, Boston Scientific, Boston, MA, United States; 1 patient, Activa RC, Medtronic, Minneapolis, MN, United States; 2 patients, Activa SC, Medtronic, Minneapolis, MN, United States; 2 patients, Percept PC, Medtronic, Minneapolis, MN, United States) (Supplementary Table S1). The average months since implantation was 15.86 ± 16.8 months. During the study, the parameters were optimized for anti-Parkinson therapy. Stimulation amperes were [right: 1.5–3.5 (2.6 ± 0.5) mA; left: 1.4–3.2 (2.5 ± 0.5) mA] and Hertz [right: 130–200 (135.9 ± 17.8) Hz; left: 130–200 (135.6 ± 16.8) Hz]. Twenty patients showed symptoms predominantly on the right side.

### Study design

**Figure 1:**
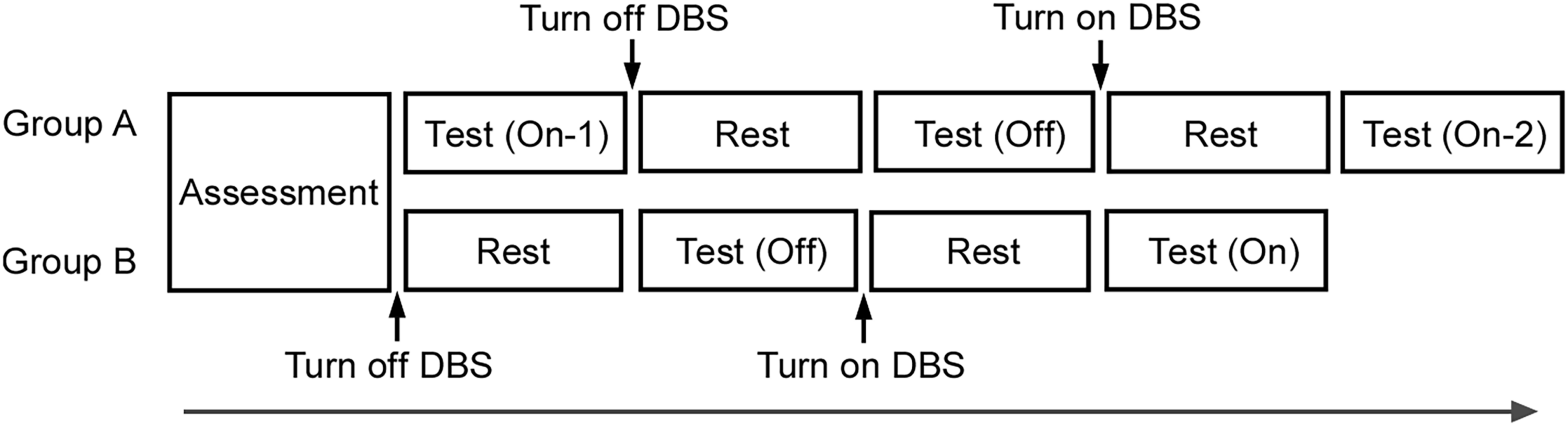
Experimental design. After conducting the assessment, in group A, the test was conducted thrice in the order of On-1, Off, and On-2. In group B, the test was conducted twice in the order of Off and On. After approximately 20 min of turning the DBS device On or Off, the next test was performed. The same five tasks were conducted in a test.

In this study, a prospective, single-blinded and within-subject repeated measures design was used to investigate and compare the effects of DBS. The participants were divided into two groups (groups A and B), in a randomized manner, to assess the effects of order and repetition on the same tasks. In group A, the test was conducted thrice in the order of On-1 (first test of On condition of STN-DBS), Off, and On-2 (second test of On condition of STN-DBS). In group B, the test was conducted two times in the order of Off and On (On and Off conditions performed once each, Figure 1). The next test session was performed approximately 20 min after switching the DBS device from On to Off or from Off to On. Each time, the neurologist confirmed whether the effects of DBS were clearly lost when DBS was switched Off or emerged when it was switched On based on the patients’ symptoms, including tremor at rest, muscle rigidity, akinesia, and postural maintenance. The same five tasks were conducted in each session. Temporal sense processing was assessed in three different tasks (time reproduction, time production, and bisection). Additionally, length production and simple reaction tasks were conducted.

### Procedures

In the time production task, the duration of the interval to be produced was presented on the monitor screen as a number of seconds for 3 s at the beginning of each trial. Patients were not informed the duration of the cue. After the number presentation disappeared from the screen, patients produced the instructed time duration by pressing the button twice at the start and end of the duration, such that the time interval between the first two and last two button presses corresponded to the required duration (Supplementary Figure S1A). The durations to be produced were 2, 3, 4, 5, 6, 7, and 8 s. Patients were not provided with feedback on their produced duration. Each duration was repeated thrice (total: 21 repetitions), with the trial order randomized and counterbalanced among participants. Data for the time production task were calculated by the duration produced compared to the specified number of seconds, expressed as a percentage.

The time bisection task comprised two phases: learning and test (Supplementary Figure S1B). In the learning phase, circles appeared on the screen for a long (8 s) or short duration (2 s). These were considered the “standard durations.” During the learning phase, each standard (long and short) was shown on the screen 10 times, for a total of 20 repetitions. In the test phase, the circles were shown for durations of 2, 3, 4, 5, 6, 7, or 8 s. In each trial, subjects were asked to indicate whether the duration shown was “closer to the short standard” or “closer to the long standard.” Each duration was repeated five times in the test phase (35 repetitions), and the trial order was randomized. Results for the bisection task were calculated as the proportion of “long” responses shown as a percentage.

The time reproduction task was conducted to examine the role of short-term memory in the sense of time duration. A circle was shown on screen for a specified duration at the beginning of each trial. After the sample disappeared from the screen, patients reproduced the circle presentation duration by pressing the button twice, one for start and another for end, so that the time interval between the two button presses corresponded to the patient’s estimate of the duration (Supplementary Figure S1C). The durations of 2, 3, 4, 5, 6, 7, and 8 s were presented in each trial. The patients had no way of knowing the actually presented duration of the circle. Each duration was repeated thrice (total: 21 repetitions), and the trial order was randomized. Results on the time reproduction task were calculated by the duration estimated by the patients compared to the actual duration, expressed as a percentage.

To examine basic motor function, a simple reaction task was conducted. Patients were instructed to press a response button using their dominant hand as soon as a figure (circle) appeared on the computer screen. The same trial was repeated thrice per session. To assess whether DBS affects spatial sense processing, we conducted the length production task. The patients were asked to move a circular figure on the computer screen 10 cm to the right in the absence of any distance measuring cue. The same trial was also repeated thrice per session. Results for the length production task were calculated as the patient’s estimate of 10 cm compared to an actual distance of 10 cm, expressed as a percentage.

The order of the three temporal tasks was randomized among patients. The simple reaction and length production tasks were done after the temporal tasks. No feedback was provided to patients in all tasks.

### Statistical analyses

To examine an effect of repetition and trial order of the same tasks, a paired *t*-test was performed to analyze differences between the On-1 and On-2 conditions in each index in group A. Using an unpaired *t*-test, we analyzed the difference between the On-2 condition in group A and that in group B, and the difference between the Off conditions of the two groups. Next, after the On condition (On-2 condition in group A and On condition in group B) and Off condition (Off condition in group A and group B) data were averaged separately, we analyzed differences between the On and Off conditions using the paired *t*-test. All tests were two-tailed. Results are shown as mean ± standard error of the mean (SEM). Statistical significance was set at adjusted *p* < 0.05. SPSS version 26 for Windows (IBM, Inc., Chicago, IL, United States) was used for the analyses.

## Results

### Time production task

**Figure 2:**
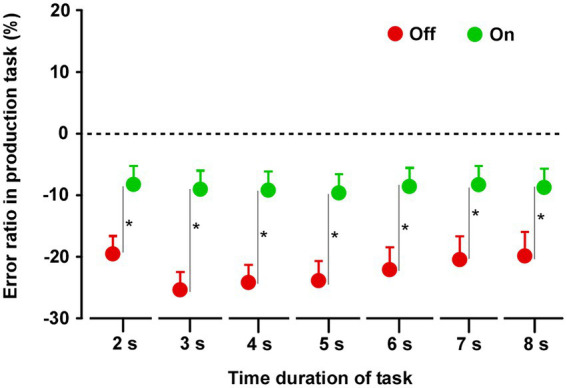
Results of the time production task. Paired *t*-tests were performed across the two groups. The duration in the Off condition was shorter than that in the On condition for all task durations. Error bars show standard error mean (SEM). Asterisks indicate significant differences (*p* < 0.0001).

In both groups, the produced time durations in the Off condition were shorter than those in the On condition (Supplementary Figure S2). In group A (*n* = 14), there was no difference between the On-1 and On-2 conditions. Furthermore, there was no difference between the On-2 condition in group A and the On condition in group B, nor was there a difference in the Off condition between the two groups. When the On condition (On-2 condition in group A and On condition in group B) and Off condition (Off condition in the groups A and B) data were averaged separately, there were significant differences between the On and Off conditions. The durations in the Off condition were significantly shorter than those in the On condition for all task durations (2, 3, 4, 5, 6, 7, and 8 s) (2 s: *t*_27_ = 3.997, *p* < 0.0001; 3 s: *t*_27_ = 7.092, *p* < 0.0001; 4 s: *t*_27_ = 7.649, *p* < 0.0001; 5 s: *t*_27_ = 8.902, *p* < 0.0001; 6 s: *t*_27_ = 8.756, *p* < 0.0001; 7 s: *t*_27_ = 8.729, *p* < 0.0001; 8 s: t_27_ = 9.955, *p* < 0.0001) (Figure 2).

### Time bisection task

**Figure 3:**
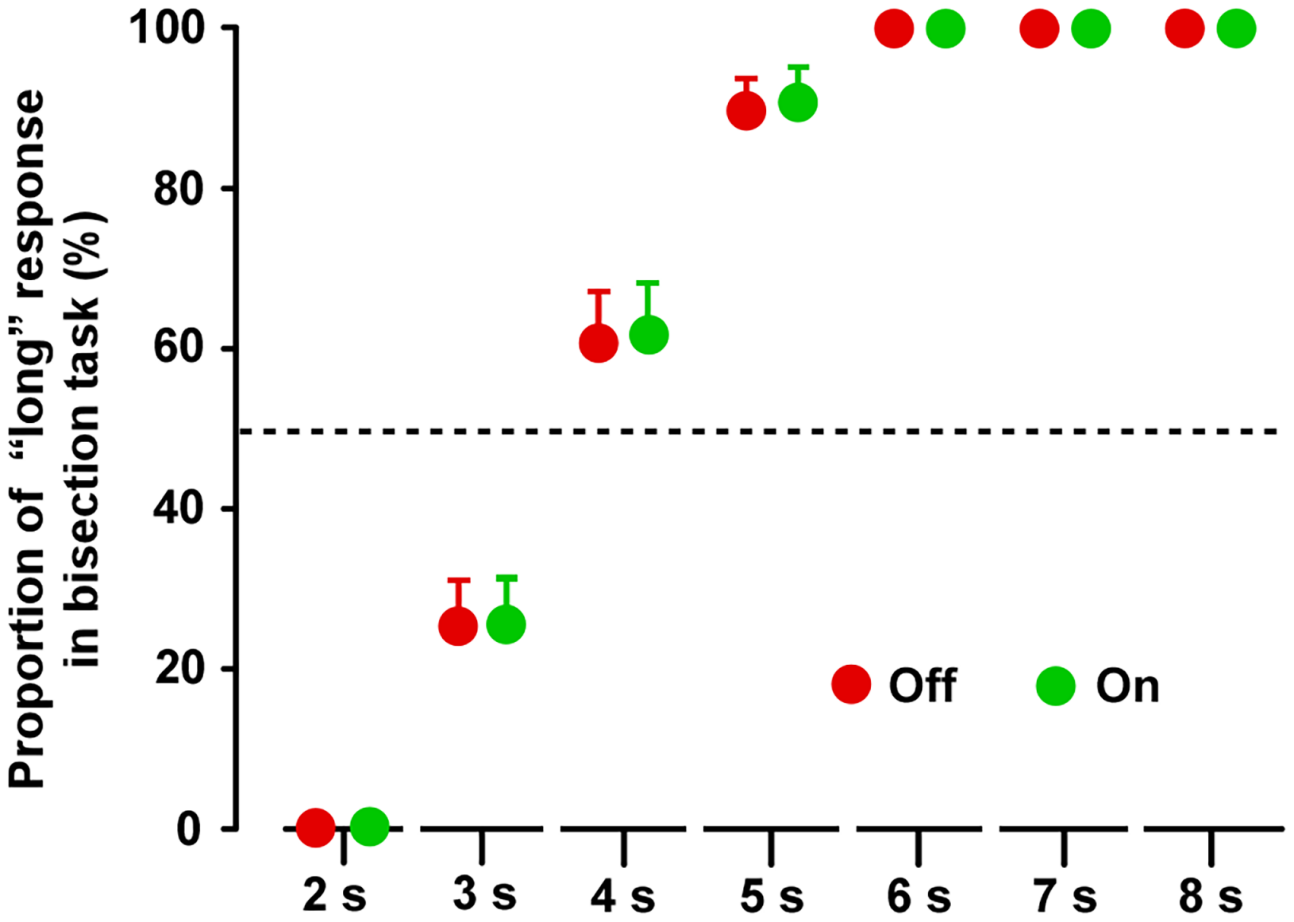
Results of the time bisection task. Across the two groups, repeated measures analysis of variance (RM-ANOVA) showed that there were no main effects of STN-DBS for all durations (2, 3, 4, 5, 6, 7, and 8 s). Error bars show SEM.

In both groups, the proportion of “long” response for the 2 s duration was 0%, and the proportion for 6, 7, and 8 s were 100%, in both the Off and On conditions (Supplementary Figure S3). In group A, there were no significant differences between the On-1 and On-2 conditions, between the On-2 condition in group A and the On condition in group B, or between the Off condition in the two groups. After the On condition and Off condition data were averaged separately, the paired *t*-test revealed no significant difference between the On and Off conditions for all task durations (2–8 s) (Figure 3).

### Time reproduction task

**Figure 4:**
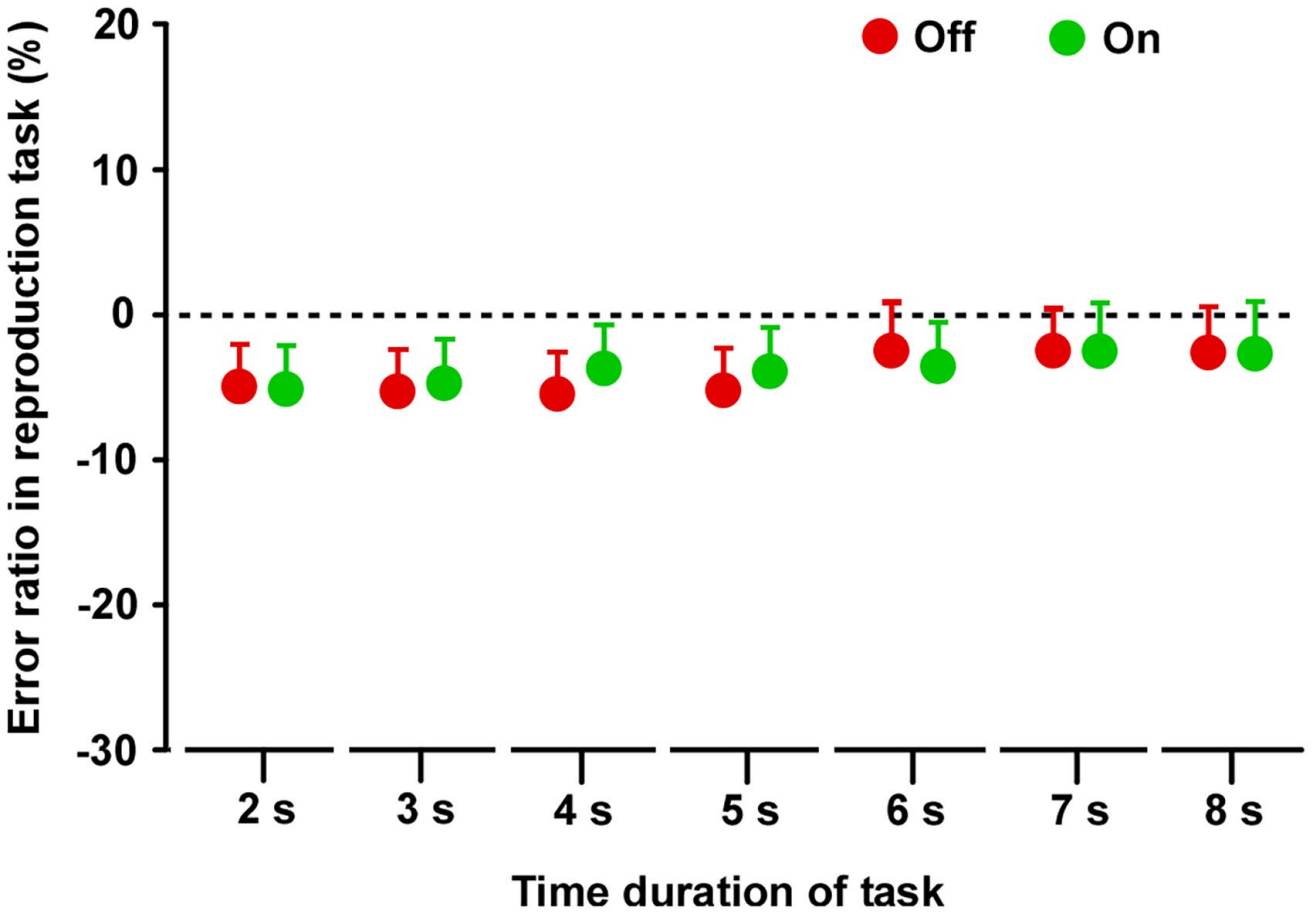
Results of the time reproduction task. Across the two groups, repeated measures analysis of variance (RM-ANOVA) showed that there were no main effects of STN-DBS for all task durations (2, 3, 4, 5, 6, 7, and 8 s). Error bars show SEM.

In both groups, there was no difference in reproduced durations between Off and On conditions (Supplementary Figure S4) on all tasks. There was no significant difference between On-1 and On-2 conditions in group A, between the On-2 condition in group A and the On condition in group B, or between the Off conditions in the two groups. Finally, there was no difference between the On and Off conditions for all task durations (2–8 s) (Figure 4).

### Length production task

**Figure 5:**
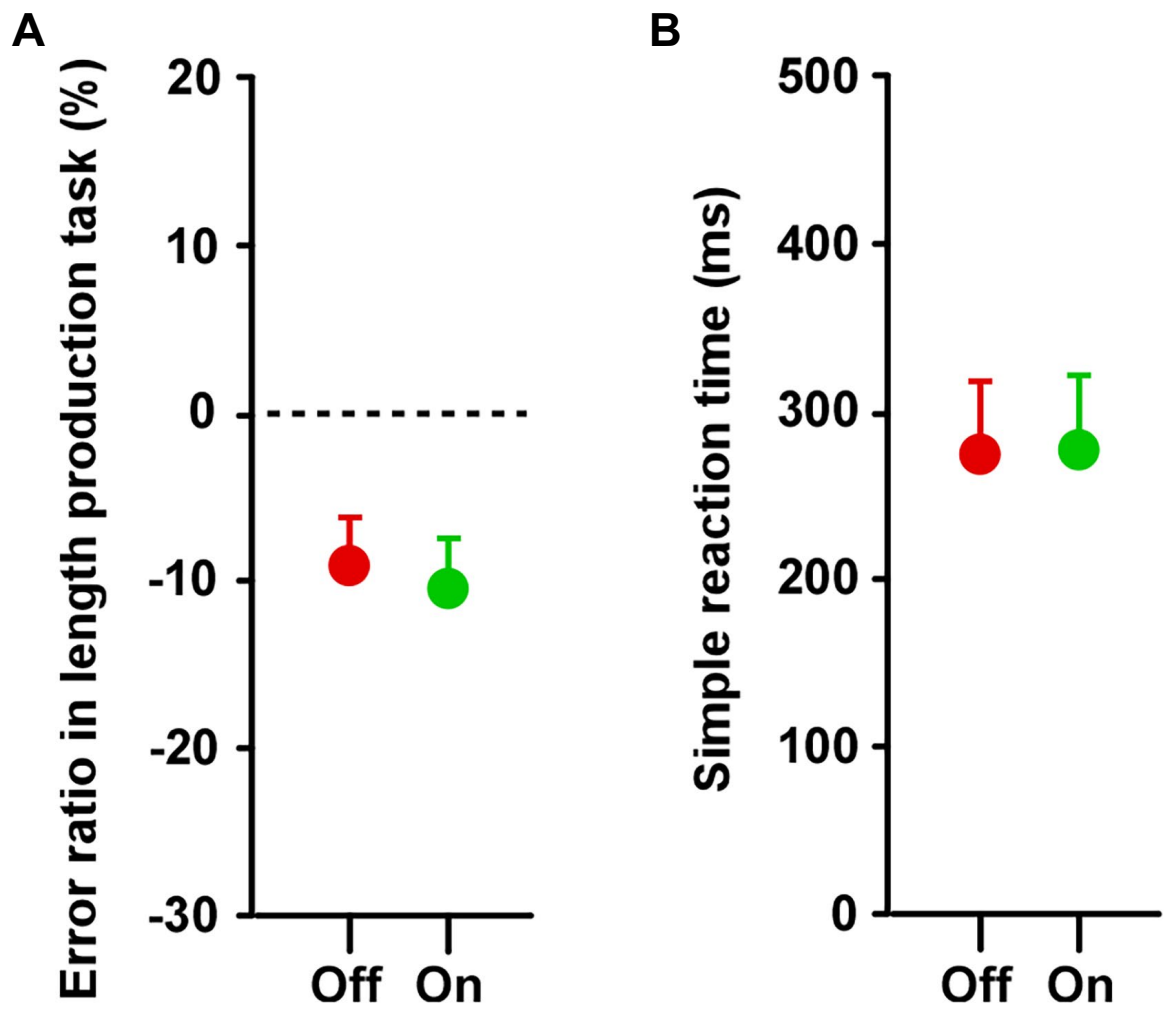
Results of **(A)** length production and **(B)** simple reaction tasks. Across the two groups, paired *t*-tests showed that there was no difference in the duration between Off and On conditions in both the length production and simple reaction tasks. Error bars show SEM.

In both groups, there was no difference in the estimated lengths between Off and On conditions (Supplementary Figure S5). In group A, there was no significant difference between On-1 and On-2 conditions, between the On-2 condition in group A and the On condition in group B, or between the Off condition in the two groups. Overall, there was no difference between the On and Off conditions (Figure 5A).

### Simple reaction task

There was no difference in reaction time for the simple reaction task between the Off and On conditions (Supplementary Figure S6). In group A, there was no significant difference between On-1 and On-2 conditions, between the On-2 condition in the group A and the On condition in group B, or between the Off condition in the two groups. There was no significant difference in reaction time between the On and Off conditions (Figure 5B).

## Discussion

The current study investigated the role of STN in temporal sensory processing especially in the sense of time, by manipulating STN function in PD patients off medication. Only the time production task showed a change with STN-DBS, with the estimate of time duration becoming shorter when STN-DBS was turned Off. In contrast, in the time bisection and time reproduction tasks, there was no difference between the Off and On conditions. These indicate that the functional manipulation of STN mainly affected the time production task, or the exteriorization (read out) of reference memory formed by experience, but not the temporal processing for time bisection and reproduction.

The bisection task requires subjects to remember long/short durations during the learning session and then judge the present time duration in reference to the learned duration during the test session, after the clock stage processing has been completed, and only engages reference memory to a small extent. Lack of effect of STN-DBS on the bisection task implies that the internal clock speed, or the “scale” used to perceive and estimate the present time duration, is not affected by STN-DBS. Rather, the difference in results among different temporal tasks can be ascribed to STN-DBS affecting the time representation in the memory stage, which may be classified into two types: reference and short-term memory. The production task would mainly require referral to and retrieval from reference memory but does not engage short-term memory (read out). In contrast, in the reproduction task, the time reproduced will be close to the veridical time duration as long as it is encoded and reproduced using the same clock; short-term memory but not reference memory in long-term memory is mainly used to perform the task. The effect of STN-DBS on the production task suggests that STN-DBS affects the reference memory processing in the memory stage (long-term memory). In contrast, lack of STN-DBS effect in the bisection task suggests that STN does not affect short-term memory processing. The effect of STN-DBS was even less evident in the reproduction task, suggesting that STN-DBS does not affect short-term memory processing. Our results thus suggest that reference and short-term memory may be affected differently during temporal processing in PD.

Meanwhile, the lack of STN-DBS effect on the length production and simple reaction tasks suggest that STN-DBS does not affect spatial representation and basic motor function. Since all the temporal tasks in this study involved button presses and were dependent on motor function, we have to differentiate whether the effect of DBS was specific to temporal processing or was rather due to its effect on basic motor function. We assumed that if PD affected basic motor function and that the main reason for the altered task performance in PD patients was the result of this or the effect of DBS on motor function, it would affect motor function to a similar degree in all temporal tasks (production, reproduction, and bisection), with similar results in all three tasks, but this was not the case. On the other hand, the fact that there was no effect of DBS On/Off in the simple reaction task suggests that, at least, the influence of DBS on basic motor function as assessed by the speed of button responses was minimal.

The basal ganglia-thalamocortical circuit comprises the direct, indirect, and hyper-direct pathways. In the direct and indirect pathways, the striatum is the input stage, whereas internal segments of the globus pallidus (GPi) and the substantia nigra pars reticulata (SNr) constitute the output stages of the basal ganglia. The indirect pathway leads from the striatum to the external segment of the globus pallidus (GPe) and STN, which in turn project to the GPi and SNr. Here the STN is positioned as an important relay nucleus (Albin et al., 1989; DeLong, 1990), receiving excitatory input from the cerebral cortex and inhibitory input from the GPe, and sends excitatory projections to the GPe, GPi, and SNr. Furthermore, *via* the hyper-direct pathway, STN receives inputs directly from the frontal areas involved in inhibition and executive control (Jahanshahi, 2013).

In PD, dopamine depletion in the striatum leads to decreased activity of neurons in the direct pathway and increased activity of neurons in the indirect pathway, resulting in increased activity of the GPi, decreased activity of the GPe, and increased activity of the STN (Miller and DeLong, 1988; Bergman et al., 1990). STN-DBS inhibits the overactivity of the STN (Breit et al., 2004). This inhibition may affect the pathways that project to the GPi and SNr and affect higher order functions, mainly the prefrontal cortex in the basal ganglia-thalamo-cortical circuit (Alexander and Crutcher, 1990; Oswal et al., 2013; Terao et al., 2021). Our findings suggest that the role of the STN in temporal sense processing may be reference to the time representation in long-term memory. Conversely, STN-DBS may have little effect on the function of perceiving the present time duration and may also have little effect on maintaining temporal short-term memory for performing the time reproduction task. Consistent with these views, a study showed in primates that L-DOPA treatment ameliorated PD signs, particularly akinesia/bradykinesia, and normalized cortically evoked responses in both the GPi and GPe, whereas STN blockade by muscimol injection ameliorated motor deficits and unmasked cortically evoked inhibition in the GPi (Chiken et al., 2021).

Based on this neural network, we may speculate how the coding of time representation in the short-and long-term is differently implemented by the basal ganglia thalamo-cortical circuit, leading to the differential effect of STN-DBS on different temporal tasks. Two memory-related brain structures are involved in temporal processing: the prefrontal cortex and the hippocampus. Honma et al. previously showed that the time scale representation for time sense, possibly in the long-term temporal memory, is abnormally shortened in PD patients (Honma et al., 2021), and this false learning of time scale learning may be consolidated by quadripulse transcranial magnetic stimulation over the prefrontal cortex (Honma et al., 2022). This may be stored in the prefrontal cortex as a form of temporal long-term memory, and may be directly affected by the functional manipulation induced by STN-DBS. Another pathway projecting from the striatum through the hippocampus to SNr bypasses STN, but projects to the thalamus and is relayed through the cortex. The time representation in short-term memory using this pathway may be less affected by PD or by the functional manipulation of STN-DBS.

This study has several limitations. The primary effect of STN DBS is on motor symptoms of PD patients (Hariz and Blomstedt, 2022). In the present study, we assessed basic motor function by using the simple reaction task and found no effect of DBS-STN on it. However, this task represents only one aspect of the overall motor symptoms. In the future, it may be necessary to use a more extensive experimental measure to assess motor symptoms, such as the UPDRS part-III for On/Off conditions in each subject. Second, it is possible that DBS-STN influenced patients’ emotional/psychological state. For example, the temporal processing may have been highly affected by the presence of unpleasant symptoms (Mella et al., 2019). Experiments with designs controlling for the DBS-STN effects on emotional/psychological state would need to be conducted. Third, all of the temporal tasks in this study were related to the visual domain. Temporal processing has been shown to have a more strong effect in the auditory domain (Wehrman and Sowman, 2021). Using a task that estimates the duration of sounds instead of visual signal might produce more robust results. Fourth, the sex of the patients in this experiment was biased for males. Since gender differences are known in time perception (Geer et al., 1964; Hancock et al., 1992), it would be important for future studies to conduct an experiment with subjects of both genders of equal sample size. Fifth, only medications related to Parkinson’s disease were discontinued in the current study. Half-lives of some medicines may be so long as to be confounding variables, and non-Parkinson’s disease-related medications could easily impact time perception. Some subjects also had comorbid symptoms or diseases other than PD, which may have also affected their time perception. Finally, the present study was necessarily performed in PD patients treated with DBS because the DBS-STN is not performed in healthy subjects. PD thus becomes the only human model, but since time can be distorted by a degenerative neurologic conditions, any conclusions are very tentative. Studies using primate models of Parkinsonism have provided insights into the mechanism of action of DBS in PD, in which STN DBS has been suggested to functionally interfere the function of the overactive STN-GPi/SNr pathway, which works to normalize the function of basal-ganglia thalamocortical pathway (Albin et al., 1989; Bergman et al., 1990; Calabresi et al., 2014; Pappas et al., 2014). However, DBS has not been studied in the context of time perception, partly since temporal sense in humans and primates may not be equated, and the size differences between rodent and primate anatomy makes it difficult to translate the findings from rodents to humans (Hardman et al., 2002). In the future, temporal sense experiments using DBS-STN in animals will be necessary.

## Conclusion

This study examined the role of STN in the temporal sense processing in the few seconds range in PD patients using three temporal processing tasks. Unlike the hundreds of milliseconds range addressed in previous study (Cope et al., 2014), STN-DBS affects temporal sense in the seconds range but only for the performance of the temporal production task (Rao et al., 2001; Meck et al., 2008). STN-DBS may enhance the function of the prefrontal cortex through the basal ganglia-thalamo-cortical circuit, and improve access to memory representation (time scale) for reading out time duration using reference memory.

## Data availability statement

The original contributions presented in the study are included in the article/Supplementary material, further inquiries can be directed to the corresponding author/s.

## Ethics statement

The studies involving human participants were reviewed and approved by the ethics committee of Juntendo University School of Medicine. The patients/participants provided their written informed consent to participate in this study.

## Author contributions

MH and YT designed the study. FS, HKa, MN, HKu, AU, HI, YS, GO, and NH recruited patients and conducted the investigation. MH, FS, HK, and TA analyzed the data. MH and YT wrote the manuscript. All authors contributed to the article and approved the submitted version.

## Funding

This study was funded by JSPS KAKENHI, Grant-in-Aid for Scientific Research (C) (JP18K03185), and Grant-in-Aid for Scientific Research on Innovative Areas (JP18H05523).

## Conflict of interest

The authors declare that the research was conducted in the absence of any commercial or financial relationships that could be construed as a potential conflict of interest.

## Publisher’s note

All claims expressed in this article are solely those of the authors and do not necessarily represent those of their affiliated organizations, or those of the publisher, the editors and the reviewers. Any product that may be evaluated in this article, or claim that may be made by its manufacturer, is not guaranteed or endorsed by the publisher.

## References

[ref1] Albin R. L. Young A. B. Penney J. B. (1989). The functional anatomy of basal ganglia disorders. Trends Neurosci. 12, 366–375. doi: 10.1016/0166-2236(89)90074-x 2479133

[ref2] Alexander G. E. Crutcher M. D. (1990). Functional architecture of basal ganglia circuits: neural substrates of parallel processing. Trends Neurosci. 13, 266–271. doi: 10.1016/0166-2236(90)90107-l, PMID: 1695401

[ref3] Atakan Z. Morrison P. Bossong M. G. Martin-Santos R. Crippa J. A. (2012). The effect of cannabis on perception of time: a critical review. Curr. Pharm. Des. 18, 4915–4922. doi: 10.2174/138161212802884852, PMID: 22716134

[ref4] Baudouin A. Vanneste S. Isingrini M. Pouthas V. (2006). Differential involvement of internal clock and working memory in the production and reproduction of duration: a study on older adults. Acta Psychol. 121, 285–296. doi: 10.1016/j.actpsy.2005.07.004, PMID: 16139783

[ref5] Bergman H. Wichmann T. DeLong M. R. (1990). Reversal of experimental parkinsonism by lesions of the subthalamic nucleus. Science 249, 1436–1438. doi: 10.1126/science.2402638 2402638

[ref6] Breit S. Schulz J. B. Benabid A. L. (2004). Deep brain stimulation. Cell Tissue Res. 318, 275–288. doi: 10.1007/s00441-004-0936-0 15322914

[ref7] Buhusi C. V. Meck W. H. (2005). What makes us tick? Functional and neural mechanisms of interval timing. Nat. Rev. Neurosci. 6, 755–765. doi: 10.1038/nrn1764, PMID: 16163383

[ref8] Calabresi P. Picconi B. Tozzi A. Ghiglieri V. Di Filippo M. (2014). Direct and indirect pathways of basal ganglia: a critical reappraisal. Nat. Neurosci. 17, 1022–1030. doi: 10.1038/nn.3743, PMID: 25065439

[ref9] Chiken S. Takada M. Nambu A. (2021). Altered dynamic information flow through the Cortico-basal ganglia pathways mediates Parkinson's disease symptoms. Cereb. Cortex 31, 5363–5380. doi: 10.1093/cercor/bhab164, PMID: 34268560 PMC8568006

[ref10] Cope T. E. Grube M. Mandal A. Cooper F. E. Brechany U. Burn D. J.. (2014). Subthalamic deep brain stimulation in Parkinsons disease has no significant effect on perceptual timing in the hundreds of milliseconds range. Neuropsychologia 57, 29–37. doi: 10.1016/j.neuropsychologia.2014.02.021, PMID: 24613477 PMC4022837

[ref11] DeLong M. R. (1990). Primate models of movement disorders of basal ganglia origin. Trends Neurosci. 13, 281–285. doi: 10.1016/0166-2236(90)90110-v 1695404

[ref12] Duchek J. M. Balota D. A. Ferraro F. R. (1994). Component analysis of a rhythmic finger tapping task in individuals with senile dementia of the Alzheimer type and in individuals with Parkinson’s disease. Neuropsychology 8, 218–226. doi: 10.1037/0894-4105.8.2.218

[ref13] Folstein M. F. Folstein S. E. McHugh P. R. (1975). "mini-mental state". A practical method for grading the cognitive state of patients for the clinician. J. Psychiatr. Res. 12, 189–198. doi: 10.1016/0022-3956(75)90026-6, PMID: 1202204

[ref14] Geer J. H. Platt P. E. Singer M. (1964). A sex difference in time estimation. Percept. Mot. Skills 19:42. doi: 10.2466/pms.1964.19.1.42 14197465

[ref15] Gibbon J. (1977). Scalar expectancy theory and Weber’s law in animal timing. Psychol. Rev. 84, 279–325. doi: 10.1037/0033-295X.84.3.279

[ref16] Gibbon J. Church R. M. Meck W. H. (1984). Scalar timing in memory. Ann. N. Y. Acad. Sci. 423, 52–77. doi: 10.1111/j.1749-6632.1984.tb23417.x 6588812

[ref17] Hancock P. A. Vercruyssen M. Rodenburg G. J. (1992). The effect of gender and time-of-day on time perception and mental workload. Curr. Psychol. 11, 203–225. doi: 10.1007/BF02686841

[ref18] Hardman C. D. Henderson J. M. Finkelstein D. I. Horne M. K. Paxinos G. Halliday G. M. (2002). Comparison of the basal ganglia in rats, marmosets, macaques, baboons, and humans: volume and neuronal number for the output, internal relay, and striatal modulating nuclei. J. Comp. Neurol. 445, 238–255. doi: 10.1002/cne.10165, PMID: 11920704

[ref19] Hariz M. Blomstedt P. (2022). Deep brain stimulation for Parkinson's disease. J. Intern. Med. 292, 764–778. doi: 10.1111/joim.13541, PMID: 35798568 PMC9796446

[ref20] Harrington D. L. Haaland K. Y. Knight R. T. (1998). Cortical networks underlying mechanisms of time perception. J. Neurosci. 18, 1085–1095. doi: 10.1523/JNEUROSCI.18-03-01085.1998, PMID: 9437028 PMC6792777

[ref21] Honma M. Kuroda T. Futamura A. Shiromaru A. Kawamura M. (2016). Dysfunctional counting of mental time in Parkinson's disease. Sci. Rep. 6:25421. doi: 10.1038/srep25421, PMID: 27146904 PMC4857080

[ref22] Honma M. Masaoka Y. Koyama S. Kuroda T. Futamura A. Shiromaru A.. (2018). Impaired cognitive modification for estimating time duration in Parkinson's disease. PLoS One 13:e0208956. doi: 10.1371/journal.pone.0208956, PMID: 30543694 PMC6292599

[ref23] Honma M. Murai Y. Shima S. Yotsumoto Y. Kuroda T. Futamura A.. (2017). Spatial distortion related to time compression during spatiotemporal production in Parkinson's disease. Neuropsychologia 102, 61–69. doi: 10.1016/j.neuropsychologia.2017.06.004, PMID: 28601527

[ref24] Honma M. Murakami H. Yabe Y. Kuroda T. Futamura A. Sugimoto A.. (2021). Stopwatch training improves cognitive functions in patients with Parkinson's disease. J. Neurosci. Res. 99, 1325–1336. doi: 10.1002/jnr.24812, PMID: 33594677

[ref25] Honma M. Saito S. Atsumi T. Tokushige S. I. Inomata-Terada S. Chiba A.. (2022). Inducing cortical plasticity to manipulate and consolidate subjective time interval production. Neuromodulation 25, 511–519. doi: 10.1111/ner.13413, PMID: 35667769

[ref26] Ivry R. B. Keele S. W. (1989). Timing functions of the cerebellum. J. Cogn. Neurosci. 1, 136–152. doi: 10.1162/jocn.1989.1.2.136 23968462

[ref27] Jahanshahi M. (2013). Effects of deep brain stimulation of the subthalamic nucleus on inhibitory and executive control over prepotent responses in Parkinson's disease. Front. Syst. Neurosci. 7:118. doi: 10.3389/fnsys.2013.00118, PMID: 24399941 PMC3872293

[ref28] Jones C. R. Claassen D. O. Yu M. Spies J. R. Malone T. Dirnberger G.. (2011). Modeling accuracy and variability of motor timing in treated and untreated Parkinson's disease and healthy controls. Front. Integr. Neurosci. 5:81. doi: 10.3389/fnint.2011.00081, PMID: 22207839 PMC3245650

[ref29] Joundi R. A. Brittain J. S. Green A. L. Aziz T. Z. Jenkinson N. (2012). High-frequency stimulation of the subthalamic nucleus selectively decreases central variance of rhythmic finger tapping in Parkinson's disease. Neuropsychologia 50, 2460–2466. doi: 10.1016/j.neuropsychologia.2012.06.017, PMID: 22749972

[ref30] Jozefowiez J. Machado A. (2013). On the content of learning in interval timing: representations or associations? Behav. Process. 95, 8–17. doi: 10.1016/j.beproc.2013.02.011 23470799

[ref31] Kagi G. Bhatia K. P. Tolosa E. (2010). The role of DAT-SPECT in movement disorders. J. Neurol. Neurosurg. Psychiatry 81, 5–12. doi: 10.1136/jnnp.2008.157370, PMID: 20019219

[ref32] Koch G. Brusa L. Caltagirone C. Oliveri M. Peppe A. Tiraboschi P.. (2004). Subthalamic deep brain stimulation improves time perception in Parkinson's disease. Neuroreport 15, 1071–1073. doi: 10.1097/00001756-200404290-00028, PMID: 15076737

[ref33] Koch G. Costa A. Brusa L. Peppe A. Gatto I. Torriero S.. (2008). Impaired reproduction of second but not millisecond time intervals in Parkinson's disease. Neuropsychologia 46, 1305–1313. doi: 10.1016/j.neuropsychologia.2007.12.005 18215403

[ref34] Lange K. W. Tucha O. Steup A. Gsell W. Naumann M. (1995). Subjective time estimation in Parkinson's disease. J. Neural Transm. Suppl. 46, 433–438. 8821079

[ref35] Martinez-Martin P. Gil-Nagel A. Gracia L. M. Gomez J. B. Martinez-Sarries J. Bermejo F. (1994). Unified Parkinson's disease rating scale characteristics and structure. The Cooperative Multicentric Group. Mov Disord. 9, 76–83. doi: 10.1002/mds.870090112 8139608

[ref36] Meck W. H. Penney T. B. Pouthas V. (2008). Cortico-striatal representation of time in animals and humans. Curr. Opin. Neurobiol. 18, 145–152. doi: 10.1016/j.conb.2008.08.002, PMID: 18708142

[ref37] Mella N. Bourgeois A. Perren F. Viaccoz A. Kliegel M. Picard F. (2019). Does the insula contribute to emotion-related distortion of time? A neuropsychological approach. Hum Brain Mapp 40, 1470–1479. doi: 10.1002/hbm.24460, PMID: 30387890 PMC6865709

[ref38] Merchant H. de Lafuente V. (2014). Introduction to the neurobiology of interval timing. Adv Exp Med Bio. 829, 1–13. doi: 10.1007/978-1-4939-1782-2_1 25358702

[ref39] Miller W. C. DeLong M. R. (1988). Parkinsonian symptomatology. An anatomical and physiological analysis. Ann. N. Y. Acad. Sci. 515, 287–302. doi: 10.1111/j.1749-6632.1988.tb32998.x 3364889

[ref40] Nambu A. (2004). A new dynamic model of the cortico-basal ganglia loop. Prog. Brain Res. 143, 461–466. doi: 10.1016/S0079-6123(03)43043-4, PMID: 14653188

[ref41] Nani A. Manuello J. Liloia D. Duca S. Costa T. Cauda F. (2019). The neural correlates of time: a meta-analysis of neuroimaging studies. J. Cogn. Neurosci. 31, 1796–1826. doi: 10.1162/jocn_a_01459, PMID: 31418337

[ref42] Nasreddine Z. S. Phillips N. A. Bedirian V. Charbonneau S. Whitehead V. Collin I.. (2005). The Montreal cognitive assessment, MoCA: a brief screening tool for mild cognitive impairment. J. Am. Geriatr. Soc. 53, 695–699. doi: 10.1111/j.1532-5415.2005.53221.x, PMID: 15817019

[ref43] O'Boyle D. J. Freeman J. S. Cody F. W. (1996). The accuracy and precision of timing of self-paced, repetitive movements in subjects with Parkinson's disease. Brain 119, 51–70. doi: 10.1093/brain/119.1.51, PMID: 8624694

[ref44] Ogden R. S. Samuels M. Simmons F. Wearden J. Montgomery C. (2018). The differential recruitment of short-term memory and executive functions during time, number, and length perception: an individual differences approach. Q J Exp Psychol (Hove) 71, 657–669. doi: 10.1080/17470218.2016.1271445, PMID: 27951752

[ref45] Oswal A. Brown P. Litvak V. (2013). Synchronized neural oscillations and the pathophysiology of Parkinson's disease. Curr. Opin. Neurol. 26, 662–670. doi: 10.1097/WCO.0000000000000034 24150222

[ref46] Oyama G. Shimo Y. Natori S. Nakajima M. Ishii H. Arai H.. (2011). Acute effects of bilateral subthalamic stimulation on decision-making in Parkinson's disease. Parkinsonism Relat. Disord. 17, 189–193. doi: 10.1016/j.parkreldis.2010.12.004, PMID: 21276745

[ref47] Pappas S. S. Leventhal D. K. Albin R. L. Dauer W. T. (2014). Mouse models of neurodevelopmental disease of the basal ganglia and associated circuits. Curr. Top. Dev. Biol. 109, 97–169. doi: 10.1016/B978-0-12-397920-9.00001-9, PMID: 24947237 PMC4639922

[ref48] Pastor M. A. Artieda J. Jahanshahi M. Obeso J. A. (1992). Time estimation and reproduction is abnormal in Parkinson's disease. Brain 115, 211–225. doi: 10.1093/brain/115.1.211, PMID: 1559155

[ref49] Rao S. M. Mayer A. R. Harrington D. L. (2001). The evolution of brain activation during temporal processing. Nat. Neurosci. 4, 317–323. doi: 10.1038/85191 11224550

[ref50] Santaniello S. Montgomery E. B. Gale J. T. Sarma S. V. (2012). Non-stationary discharge patterns in motor cortex under subthalamic nucleus deep brain stimulation. Front. Integr. Neurosci. 6:35. doi: 10.3389/fnint.2012.00035, PMID: 22754509 PMC3385519

[ref51] Sasaki F. Oyama G. Sekimoto S. Nuermaimaiti M. Iwamuro H. Shimo Y.. (2021). Closed-loop programming using external responses for deep brain stimulation in Parkinson's disease. Parkinsonism Relat. Disord. 84, 47–51. doi: 10.1016/j.parkreldis.2021.01.023, PMID: 33556765

[ref52] Shi Z. Church R. M. Meck W. H. (2013). Bayesian optimization of time perception. Trends Cogn. Sci. 17, 556–564. doi: 10.1016/j.tics.2013.09.009 24139486

[ref53] Smith J. G. Harper D. N. Gittings D. Abernethy D. (2007). The effect of Parkinson's disease on time estimation as a function of stimulus duration range and modality. Brain Cogn. 64, 130–143. doi: 10.1016/j.bandc.2007.01.005, PMID: 17343966

[ref54] Spencer R. M. Ivry R. B. (2005). Comparison of patients with Parkinson's disease or cerebellar lesions in the production of periodic movements involving event-based or emergent timing. Brain Cogn. 58, 84–93. doi: 10.1016/j.bandc.2004.09.010 15878729

[ref55] Tai C. H. (2022). Subthalamic burst firing: a pathophysiological target in Parkinson's disease. Neurosci. Biobehav. Rev. 132, 410–419. doi: 10.1016/j.neubiorev.2021.11.044, PMID: 34856222

[ref56] Teki S. Gu B. M. Meck W. H. (2017). The persistence of memory: how the brain encodes time in memory. Curr. Opin. Behav. Sci. 17, 178–185. doi: 10.1016/j.cobeha.2017.09.003, PMID: 29915793 PMC6004118

[ref57] Terao Y. Honma M. Asahara Y. Tokushige S. I. Furubayashi T. Miyazaki T.. (2021). Time distortion in parkinsonism. Front. Neurosci. 15:648814. doi: 10.3389/fnins.2021.648814, PMID: 33815049 PMC8017233

[ref58] Tokushige S. I. Matsuda S. I. Oyama G. Shimo Y. Umemura A. Sasaki T.. (2018). Effect of subthalamic nucleus deep brain stimulation on visual scanning. Clin. Neurophysiol. 129, 2421–2432. doi: 10.1016/j.clinph.2018.08.003, PMID: 30292079

[ref59] Tossici-Bolt L. Hoffmann S. M. Kemp P. M. Mehta R. L. Fleming J. S. (2006). Quantification of [123I]FP-CIT SPECT brain images: an accurate technique for measurement of the specific binding ratio. Eur. J. Nucl. Med. Mol. Imaging 33, 1491–1499. doi: 10.1007/s00259-006-0155-x, PMID: 16858570

[ref60] Wehrman J. Sowman P. (2021). Oddball onset timing: little evidence of early gating of oddball stimuli from tapping, reacting, and producing. Atten. Percept. Psychophys. 83, 2291–2302. doi: 10.3758/s13414-021-02257-6, PMID: 33723728 PMC7959674

[ref61] Wichmann T. Soares J. (2006). Neuronal firing before and after burst discharges in the monkey basal ganglia is predictably patterned in the normal state and altered in parkinsonism. J. Neurophysiol. 95, 2120–2133. doi: 10.1152/jn.01013.2005, PMID: 16371459

[ref62] Wild-Wall N. Willemssen R. Falkenstein M. Beste C. (2008). Time estimation in healthy ageing and neurodegenerative basal ganglia disorders. Neurosci. Lett. 442, 34–38. doi: 10.1016/j.neulet.2008.06.069, PMID: 18602449

[ref63] Wojtecki L. Elben S. Timmermann L. Reck C. Maarouf M. Jorgens S.. (2011). Modulation of human time processing by subthalamic deep brain stimulation. PLoS One 6:e24589. doi: 10.1371/journal.pone.0024589, PMID: 21931767 PMC3171456

[ref64] Zhang J. Nombela C. Wolpe N. Barker R. A. Rowe J. B. (2016). Time on timing: dissociating premature responding from interval sensitivity in Parkinson's disease. Mov. Disord. 31, 1163–1172. doi: 10.1002/mds.26631, PMID: 27091513 PMC4988382

