# Supplementary material for: Role of the subthalamic nucleus in perceiving and estimating the passage of time
Source: Front Aging Neurosci. 2023 Mar 2;15:1090052. doi: 10.3389/fnagi.2023.1090052 (PMC10017994; doi:10.3389/fnagi.2023.1090052)
Supplement: Supplementary file 2 [file Image_1.pdf]

Supplementary Information

Role of the subthalamic nucleus in perceiving and estimating the passage of time

Motoyasu Honma, Fuyuko Sasaki, Hikaru Kamo, Maierdanjiang Nuermaimaiti, Hitoshi Kujirai, Takeshi Atsumi, Atsushi Umemura, Hirokazu Iwamuro, Yasushi Shimo, Genko Oyama, Nobutaka Hattori, Yasuo Terao

Supplementary Figs. 1-6

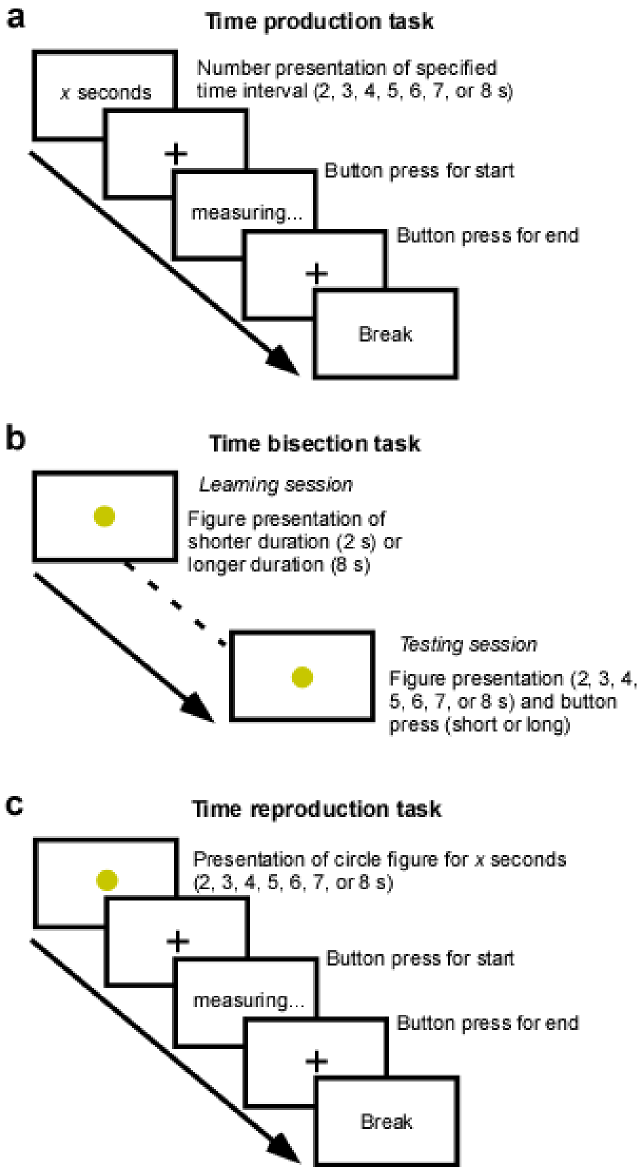

Supplementary Fig. 1. Procedures of time tasks. **a)** In the time production task, the duration of the interval to be produced was presented on the monitor screen as a number of seconds for 3 s at the beginning of each trial. After the number presentation disappeared from the screen, patients produced the instructed time duration by pressing the button twice at the start and end of the duration, such that the time interval between the first two and last two button presses corresponded to the required duration. **b)** The time bisection task comprised two phases: learning and test. In the learning phase, circles appeared on the screen for a long (8 s) or short duration (2 s). These were considered the "standard durations." During the learning phase, each standard (long and short) was shown on the screen 10 times, for a total of 20 repetitions. In the test phase, the circles were shown for durations of 2, 3, 4, 5, 6, 7, or 8 s. In each trial, subjects were asked to indicate whether the duration shown was "closer to the short standard" or "closer to the long standard". **c)** The time reproduction task was conducted to examine the role of short-term memory in the sense of time duration. A circle was shown on screen for a specified duration at the beginning of each trial. After the sample disappeared from the screen, patients reproduced the circle presentation duration by pressing the button twice, one for start and another for end, so that the time interval between the two button presses corresponded to the patient's estimate of the duration. The durations of 2, 3, 4, 5, 6, 7, and 8 s were presented in each trial. The patients had no way of knowing the actually presented duration of the circle.

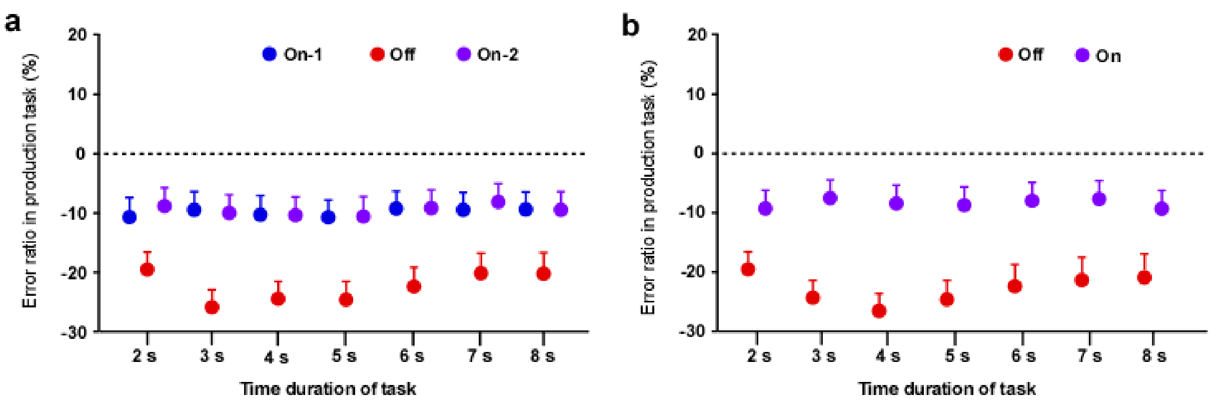

Supplementary Fig. 2. Results of time production task in the group A (panel **a**, n = 14) and group B (panel **b**, n = 14).

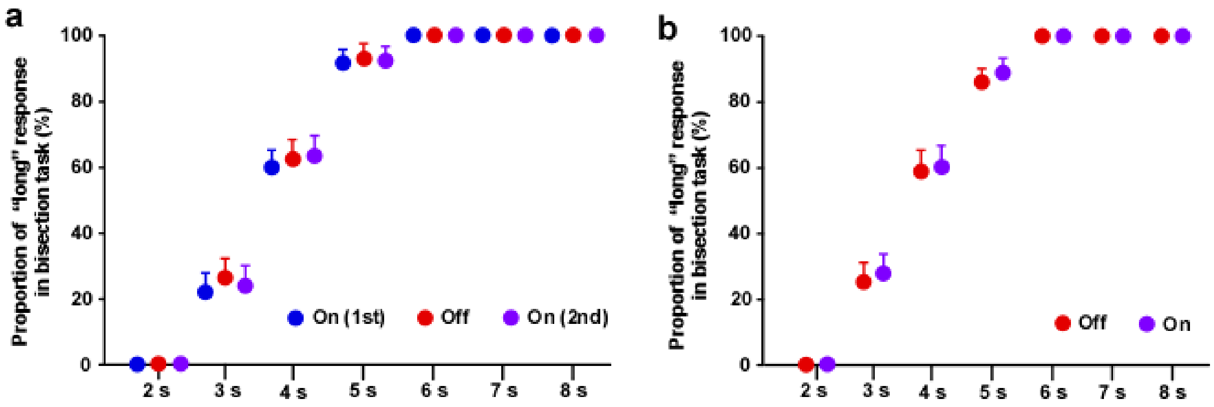

Supplementary Fig. 3. Results of time bisection task in the group A (panel **a**, n = 14) and group B (panel **b**, n = 14).

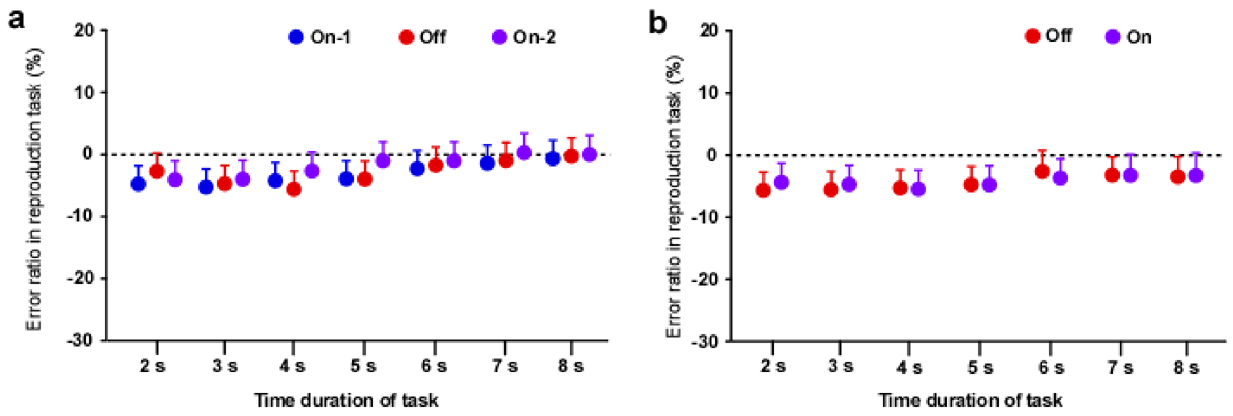

Supplementary Fig. 4. Results of time reproduction task in the group A (panel **a**, n = 14) and group B (panel **b**, n = 14).

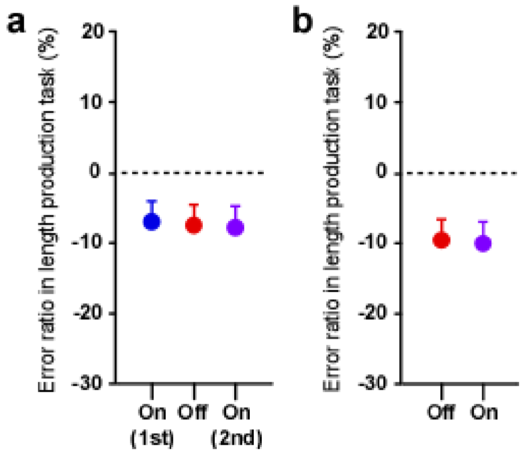

Supplementary Fig. 5. Results of length production task in the group A (panel **a**, n = 14) and group B (panel **b**, n = 14).

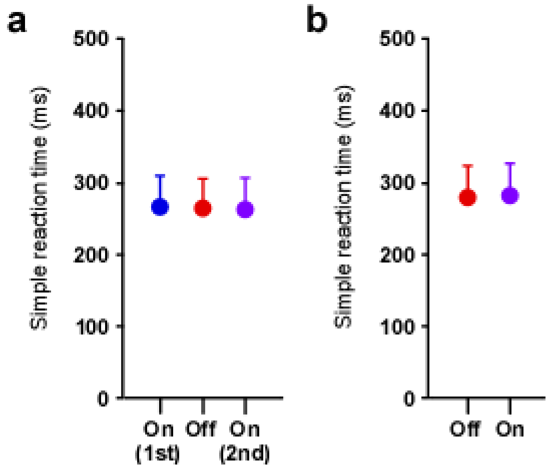

Supplementary Fig. 6. Results of length production task in the group A (panel **a**, n = 14) and group B (panel **b**, n = 14).
